# Searching for Potential Markers of Glomerulopathy in Urine by HS-SPME-GC×GC TOFMS

**DOI:** 10.3390/molecules26071817

**Published:** 2021-03-24

**Authors:** Tomasz Ligor, Joanna Zawadzka, Grzegorz Strączyński, Rosa M. González Paredes, Anna Wenda-Piesik, Ileana Andreea Ratiu, Marek Muszytowski

**Affiliations:** 1 Department of Environmental Chemistry and Bioanalytics, Faculty of Chemistry, Nicolaus Copernicus University, 87-100 Toruń, Poland; 2 Interdisciplinary Centre of Modern Technologies, Nicolaus Copernicus University, 87-100 Toruń, Poland; andreea_ratiu84@yahoo.com; 3 Department of Nephrology, Diabetology and Internal Medicine, Nicolaus Copernicus University, Rydygier Hospital, 87-100 Toruń, Poland; as.zawadzka@gmail.com (J.Z.); marek.muszytowski@gmail.com (M.M.); 4 USL Ltd., 43-110 Tychy, Poland; grzegorz.straczynski@usl.com.pl; 5 Department of Analytical Chemistry, Nutrition and Food Sciences, University of Salamanca, 37008 Salamanca, Spain; rosamgonzal@usal.es; 6 Department of Plant Growth Principles and Experimental Methods, UTP University of Science and Technology, 85-796 Bydgoszcz, Poland; apiesik@utp.edu.pl; 7 “Raluca Ripan” Institute for Research in Chemistry, Babes-Bolyai University, 30 Fantanele, RO-400239 Cluj Napoca, Romania

**Keywords:** volatile organic compounds, urine analysis, comprehensive two-dimensional gas chromatography, kidney diseases

## Abstract

Volatile organic compounds (VOCs) exiting in urine are potential biomarkers of chronic kidney diseases. Headspace solid phase microextraction (HS-SPME) was applied for extraction VOCs over the urine samples. Volatile metabolites were separated and identified by means of two-dimensional gas chromatography and time of flight mass spectrometry (GC × GC TOF MS). Patients with glomerular diseases (*n* = 27) and healthy controls (*n* = 20) were recruited in the study. Different VOCs profiles were obtained from patients and control. Developed methodology offers the opportunity to examine the metabolic profile associated with glomerulopathy. Four compounds found in elevated amounts in the patients group, i.e., methyl hexadecanoate; 9-hexadecen-1-ol; 6,10-dimethyl-5,9-undecadien-2-one and 2-pentanone were proposed as markers of glomerular diseases.

## 1. Introduction

Urine contains a multitude of organic substances, mainly products of metabolism, the majority including nitrogenous compounds. Thus, urine is also a rich source of volatile organic metabolites. For centuries organoleptic analysis of urine facilitated diagnosing illnesses, the most characteristic examples being the specific odor of urine present in diabetes and urinary tract infections. At that time, however, there were no methods that made it possible to ascertain what was responsible for the particular smell of the diseases. Shirasu et al. reviewed odoriferous compounds which are identified in urine, breath, sweat and other human secretions of ill patients and they observed to which diseases they were related [1]. A detailed study on characterization of odor active compounds in urine was conducted by Wagenstaller and coworkers. They evaluated urine samples by means of two-dimensional gas chromatographic system combined with mass spectrometry and sniffing technique [2]. At present, urine analysis constitutes an important element of medical diagnosis. However, currently used diagnostic tests do not provide information on volatile organic compounds (VOCs) present in urine, except for ketone bodies. Developments in gas chromatography and mass spectrometry as well as in sample preparation techniques naturally led to growing interest in determining VOCs in urine. The importance of VOCs for clinical diagnosis was reviewed as early as in 1981 [3]. One of the earliest applications of GC-MS in urine analysis was detection and quantitation of trimethylamine in urine of patients with fish odor syndrome [4]. Mills et al. used GC-MS and SPME for profiling VOCs in urine collected from patients with ketoacidosis, homocystinuria, hepatitis as well as from healthy persons [5]. Smith et al. identified the total of 92 substances in samples coming from 24 healthy males [6]. More recently, de Lacy Costello et al. reviewed and classified 1840 VOCs secreted from a healthy human body. They reported 279 volatiles which are identified in human urine [7]. Silva et al. used GC MS and SPME to study volatile metabolites in urine which are potentially important as cancer biomarkers. Samples were collected from a group of 33 cancer patients and 21 healthy individuals. The authors found 82 different VOCs in the group of cancer patients and in the control group [8]. Santos and coworkers analyzed ketones in urine samples to discriminate between lung cancer patients and healthy controls [9,10]. Comprehensive two-dimensional gas chromatography-time of flight mass spectrometry (GC × GC TOFMS) is a powerful tool which has been successfully used in metabolomics and biomarker discovery. This technique offers high resolution, ordered structure of chromatograms and high peak capacity and is efficient in urine analysis. GC × GC quadrupole MS or TOFMS has been used to determine anabolic steroids and their metabolites in human urine samples [11,12,13], to quantify salvinorin A in urine [14] and to detect acidic compounds in children’s urine [15]. Among the most detailed studies were those conducted by Rocha et al. exploring human urine metabolomics. They applied GC × GC-TOFMS and SPME to study VOCs in the urine headspace of healthy persons and detected ca. 700 compounds in each sample of which 294 were tentatively identified [16].

A variety of renal injuries may lead to chronic kidney diseases (CKD) [17], which is a group of pathologies, where renal excretion is chronically compromised. Most often, CKD is irreversible and progressive. Patients with end-stage kidney diseases need renal replacement therapy (RRT) such as kidney transplant or dialysis. Worldwide, about 2 million people are receiving RRT [18,19]. Numerous inflammatory and non-inflammatory diseases affect the renal glomerulus and lead to glomerular kidney diseases [20,21]. However, searching for specific VOCs in urine can be promising way to find biomarkers of glomerular diseases. The aim of the study was method development based on SPME extraction and GC × GC TOFMS analysis of VOCs from human urine. Consequently, our research work focused on identification of VOCs in urine of patients with glomerular diseases and healthy controls. We developed SPME and GC × GC TOF MS method for extraction and analysis of volatiles. An automatic method of chromatographic data processing was applied. The ability of the developed method to differentiate between the two investigated groups of subjects was proved and with it the usefulness of GC × GC TOFMS in search for glomerulopathy-specific substances in urine was demonstrated as well.

## 2. Results

### 2.1. Fiber Selection

**Figure 1:**
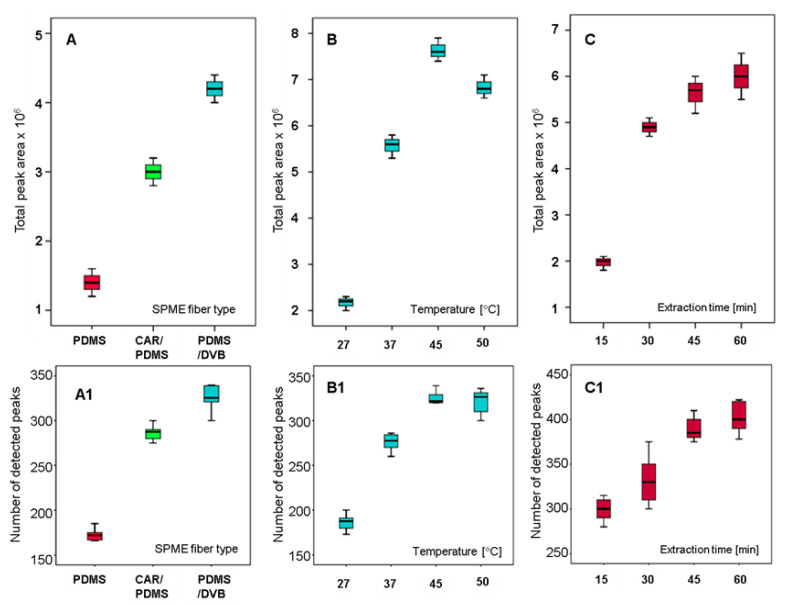
Optimization of extraction parameters, including comparison of PDMS/DVB, Carboxen/PDMS and PDMS coatings for extraction of VOC (part (**A**,**A1**)), different extraction temperatures (part (**B**,**B1**)) and extraction time (part (**C**,**C1**)). The boxplots in the upper were drawn according with total peak area of extracted VOCs (subfigures **A**–**C**), while the bottom box plots represent the number of extracted VOCs (subfigures **A1**–**C1**). Optimization of extraction parameters, including comparison of Polydimethylsiloxane/Divinylbenzene (PDMS/DVB), Carboxen/PDMS (CAR/PDMS), Polydimethylsiloxane (PDMS) for extraction of VOC (part **A**), different extraction temperatures (part **B**) and extraction time (part **C**). The boxplots in the upper were drawn according with total peak area of extracted VOCs (subfigures **A**–**C**), while the bottom box plots represent the number of extracted VOCs (subfigures **A1**–**C1**).

Three SPME fibers with different coatings-PDMS, CAR/PDMS and PDMS/DVB-were evaluated in terms of number of peaks obtained and identified as compounds, peak area and reproducibility. All fibers were exposed to the sample headspace for 30 min of incubation and 30 min of extraction, while temperature was 45 °C. As presented in Figure 1A, the PDMS/DVB fiber provided the highest extraction efficiency, since the total peak area was at the level of 4.2 × 10^6^ and RSD 10.1% was obtained with this coating. CAR/PDMS provided total peak area at the level of 3.0 × 10^6^ and RSD 12.2%. The lowest extraction efficiency was observed with the PDMS coating, with total peak area 1.4 × 10^6^ and RSD 8.4%. Thus, the PDMS/DVB fiber was selected as the SPME fiber for the analysis of the volatile compounds of urine. In terms of number of the peaks extracted, PDMS/DVB extracted more peaks (324 ± 11) than the other 2 fibers (288 ± 7 in case of CAR/PDMS and 173 ± 6 in case of PDMS), as presented in Figure 1A1. Figure 1 was created using IBM SPSS Statistics 21. The center lines of the boxes represent the mean; boxes represent mean ± SD, while whiskers represent min–max values.

### 2.2. Extraction Temperature

With the use of the PDMS/DVB fiber, 30 min of incubation time, 30 min of extraction time and with 10 mL aliquots of the same urine from a healthy volunteer, the effect of the extraction temperature was studied at 27, 37, 45 and 50 °C (Figure 1B). It was observed that both signal areas and detected number of the peaks increased gradually with increasing the temperature from 27 °C up to 45 °C. At 50 °C, the signal areas of most of the peaks decreased, while the number of the peaks remained constant with the number detected at 45 °C (Figure 1B1). According to these results and with the aim of preventing degradation of the sample at high temperature, 45 °C was chosen as the optimum value of extraction temperature. Our results regarding temperature optimization are in agreement with the results obtained by other authors. For example, Monteiro et al. applied SPME and GC-MS to the analysis of renal carcinoma patients’ urine. The authors carried out a detailed optimization of the SPME extraction process, considering SPME sorbents, urine sample pH, extraction time and temperature, etc. It was temperature that had the greatest influence on the obtained results, followed by extraction time and salt addition [10].

### 2.3. Extraction Time

The influence of the extraction time was studied as the last step of optimization. Incubation time was 30 min, followed by PDMS/DVB fiber exposure to the urine headspace at 45 °C for 15, 30, 45 and 60 min. We observed an increasing trend in the signal areas and peaks number that occurred from minute 15 to 45 (Figure 1C). In case of signal area, the very noticeable increasing trend was from 15 min to 30 min, while in case of peaks number the highest increase was from 30 min (325 peaks) to 45 min (390 peaks). After 60 min of extraction the increasing trend was insignificant in case of both signal areas and peaks number. These results showed that 45 min of the extraction time was not enough to reach the equilibrium. However, the extension of the extraction time to 60 min caused an increase in the extraction efficiency by only 5%. Similar situation was observed in case of the number of the peaks. Thus, 45 min was selected as an adequate value of extraction time.

Random variability of these signals was evaluated as well. For the whole experiment the extractions of VOCs from human urine samples were performed in triplicate, except three cases when patients did not provide a sufficient amount of samples. Repeatability was satisfactory, with RSD values lower than 7.1% for each urine sample. For variability investigation, 6 urine samples were kept in the freezer. The samples were defrosted and analyzed 45 days after sample taking at hospital sampling. Samples were analyzed by means of SPME and GC × GC TOF MS. The RSD of the measurements (*n* = 18) performed after 45 days of sample storage was in the range 6.1−9.2%.

### 2.4. Identification

The identification of volatile metabolites was performed on the basis of similarity of measured mass spectra to MS libraries (match factor > 900) and signal-to-noise (S/N) ratio (>10). For this purpose, the mass spectrum of each compound was automatically matched to those in MS libraries Wiley 9-th Ed./NIST 2011.The unique mass for each peak was chosen by the software algorithm and was used for peak area calculations. The automatically identified substances were manually verified in order to remove artefacts (mainly contaminants, silicones, column bleed, plasticizers, etc.) and compared with literature data describing the substances detected in urine headspace. For urine normalization, we selected patients and healthy persons for experiments, if the specific gravity of urine ranged from 1010 to 1030.

### 2.5. Statistical Analyses

**Figure 2:**
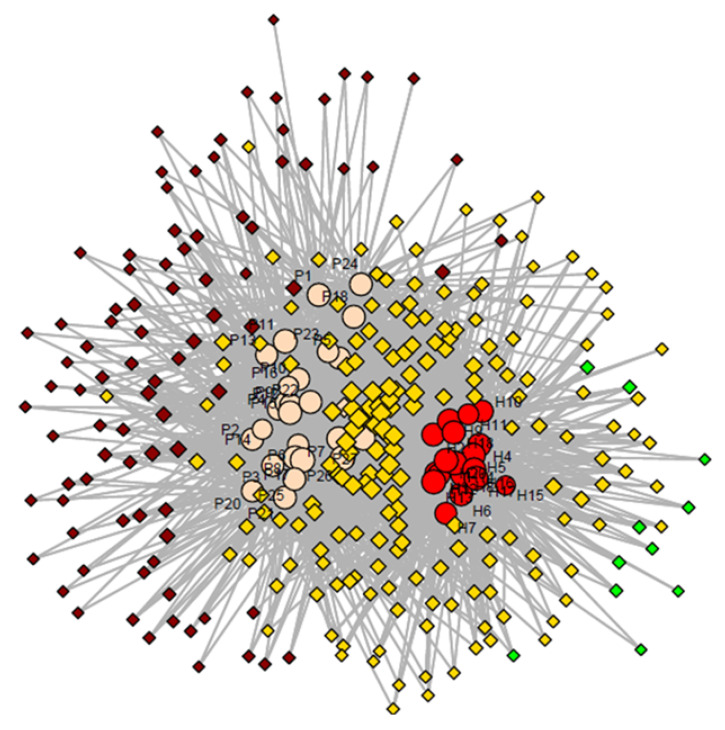
Network analyses presenting separation between the two investigated groups based on the VOCs specific for patients with glomerular diseases (brown diamonds), VOCs common between groups (yellow diamonds) and VOCs characteristic for healthy group (green diamonds), where H 1 to 20 represents the number of healthy control and P 1 to 27 the number of patients.

The peak areas of identified substances were used to build the data matrix for chemometric analyses. The dataset representing distributions of 282 investigated compounds in urine was used to build the network analysis model (Figure 2). These were all the components that appear in more than 5 samples for a given group. Consequently, based on the obtained profiles and using the incidence of the peaks, a network analysis was created in order to obtain a preliminary exploration of the data. R studio with console (version 3.6.3, Boston, MA, USA) was used for network analyses. The applied model successfully separated the two investigated groups, by leading into the formation of two cluster groups (group of diseased patients including subjects P1 to P27, clustered into the left part and group of healthy controls, represented by numbers H1 to H20, in the right down part), as presented in Figure 2. In addition, VOCs detected just in diseased patients have been dispersed around the patients group, common components were located mostly between the two groups, while VOCs detected just in healthy subjects were scattered into the right-up part (green diamonds). Regarding the number of VOCs used in network analysis, 11 were specific for healthy group, 90 for diseased group, while 181 VOCs were common between the groups. We assume that most of compounds presented in Figure 2 are endogenous generated by the organism, as a normal process of metabolism or as a response to the pathology. However, parts of VOCs are exogenous absorbed by the organism from the environment and eliminated through urine. High variability in detected VOCs coming from different subjects was found. Such phenomenon is related to several factors, among which diet, living style, personal habits, etc.

**Figure 3:**
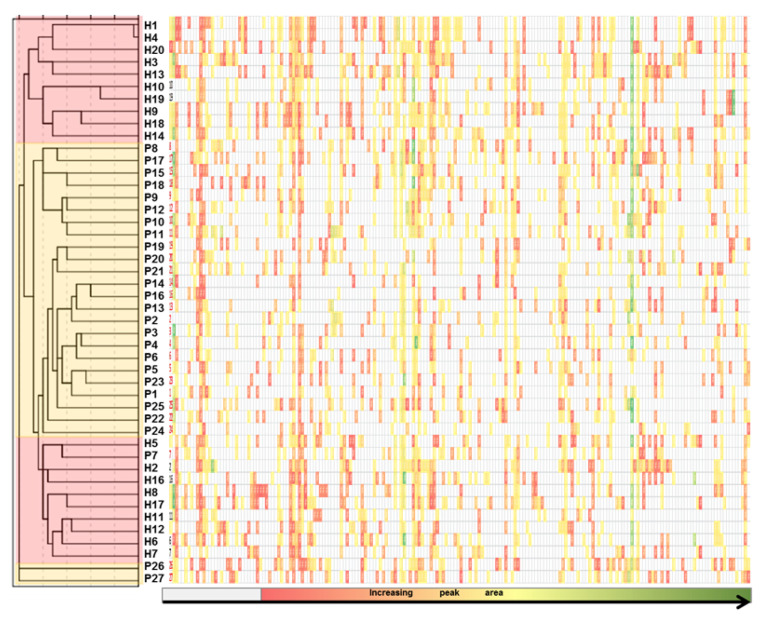
Heat map combined with dendrogram presenting hierarchical clusters segregation according with patient and control groups, where H 1to 20 represents the number of healthy control and P 1 to 27 the number of patients.

In the next step VOCs areas were used and hierarchical clusters analyses (nearest neighbor method) based on Squared Euclidean distance were created. The heat map color code, grey-red-yellow-green, is according with increasing value of peaks area, from absence (o value, highlighted in grey) to the highest area (green). Cluster segregation according with patients and control groups was obtained, as presented in Figure 3. Nevertheless, healthy group clustered in two groups that uncompressed between the diseases group. The patients P 26 and 27, expressed different characteristics and fused together in one cluster with similar distance level at the end of the dendrogram.

The peak areas of identified substances were finally used to build the data matrix for chemometric analyses, in attempt to obtain data set reduction. The aim was to search for some VOCs with discriminative features, able to be used as biomarkers of glomerular diseases. From the whole dataset representing distributions of the 146 VOCs found in urine that could be analyzed by ANOVA, thirteen compounds varied quantitatively between subjects with glomerular diseases and healthy controls (*p* < 0.05) and two at the level 0.05 < *p* < 0.1 that can be also acceptable for screening study in life science. Based on the grouping of volatile compounds using the k-mean method, all subjects with glomerular diseases had elevated level of 40 compounds (Table 1).

Generally, the significant effect was obtained for a number of 15 compounds, namely: cyclohexanone; 3-ethylcyclopentanone; 3-hexanone; 3-heptanone; methyl hexadecanoate; 9-hexadecen-1-ol; 3-methyl-2-pentanone; 6,10-dimethyl-5,9-undecadien-2-one; 2-pentanone; acetophenone; 2-methoxy-4-vinylphenol; 1-decanol; N-acetylpyrrole; 6-methylhept-5-en-2-one; dimethyl sulfone, presented in Table 1.

They play opposite role then biomarkers, while the compounds: 1, 4, 5, 6, 7, 8, 9, 10, 14, 16, 19, 21, 22, 24, 26, 27, 30, 31, 34, 35, 36, 37, 38 and 39 represent the moderate, (in ANOVA p-value is below 0.05). However, all classified compounds are presented in Table 1 with the information resulting on the F statistic.

**Figure 4:**
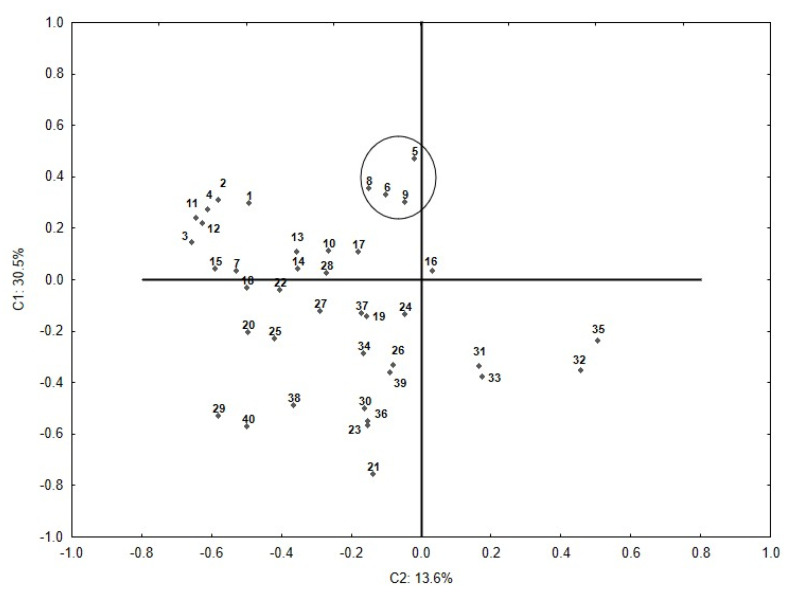
Principal component analyses (PCA) used to display variation between statistically significant determined VOCs, where: 1. cyclohexanone; 2. 3-ethylcyclopentanone; 3. 3-hexanone; 4. 3-heptanone; 5. methyl hexadecanoate; 6. 9-hexadecen-1-ol; 7. 3-methyl-2-pentanone; 8. 6,10-dimethyl-5,9-undecadien-2-one; 9. 2-pentanone; 10. acetophenone; 11. 2-methoxy-4-vinylphenol; 12. 1-decanol; 13. N-acetylpyrrole; 14. 6-methylhept-5-en-2-one; 15. dimethyl sulfone; 15. dimethyl sulfone; 16. 1-tetradecanol, 17. 4-heptanone; 18. benzaldehyde; 19. 2-nonanone; 20. 5-methyl-3-hexanone; 21. dimethyl trisulfide; 22. 2-aminobenzaldehyde; 23. 3-methylcyclopentanone; 24. hexanal; 25. 1-octanol; 26. benzeneacetaldehyde; 27. 2,5-dimethylpyrazine; 28. nonanal; 29. 9-octadecen-1-ol; 30. indole; 31. theaspirane; 32. benzonitrile; 33. 2-heptanone; 34. 4-methylphenol; 35. phenol; 36. decanal; 37. 1-methyl-4-(1-methylethenyl)-benzene; 38. N-phenylformamide, 39. ethyl acetate; 40. octanal.

Principal component analyses (PCA) were used to display total variation in the meaning of two main components results presented in Table 1 and Table 2. Contribution of individual compounds to the C1 explained 30.5% of variance. As can be observed the VOCs that loaded most positive strength on 1st components are: 5, 6, 8, 9 and they had the significant status as biomarkers, displaying higher and significant concentration in urine coming from diseased persons (they are marked in the circle). The compounds 31, 32, 33 and 35 clustered positively in C2 gathering 13.6% of total variance (Figure 4) and are both speared by the others with higher concentration in the healthy than in diseased group.

In Table 2, the VOCs are listed in the order from the highest to the lowest level of significance at which they were present in urine samples. They were obtained by the standardization of the total matrix, in order to divide the VOCs into significantly distinct groups. All the components in Table 2 were classified based on the grouping of volatile compounds using the k-mean method. The method shows that all persons with glomerular diseases had elevated level of all 40 compounds presented in Table 2.

Additionally, the compounds that present a decreasing trend can be treated as secondary chemo indicators of this category of diseases. Discrepancies between the results of statistical analyses in this respect (ANOVA and grouping of k-means) result from the fact that the tested group was a statistically small sample.

## 3. Discussion

On urine sample chromatograms, varying numbers of particular chromatographic signals were observed from 100 to 250 peaks coming from different substances present in the samples. Moreover, we observed variations in the number of samples in which certain compounds were detected. This phenomenon occurred regardless of which group the samples were collected, from the patients or healthy control. Such a large number of varied compounds posed a significant difficulty in classifying the substances and selecting potential biomarkers. This may result from a large number of factors influencing biosynthesis of volatile metabolites (metabolic pathways, genetic differences, consumed food, age, sex, physiological state, addictions, etc.). Substances present in urine can be divided into characteristic chemical groups such as ketones, aldehydes, hydrocarbons, volatile sulfur compounds, heterocycles, alcohols, phenols and terpenes. The substances most numerously represented were ketones. They amounted to almost 46% of all volatile substances identified in the samples from healthy volunteers and 49% of those found in samples from ill persons. Ketones are the products of metabolism and result from oxidation of secondary alcohols and fatty acids. Part of ketones can be also of dietary origin [6]. The most frequently observed ketones included acetone, acetophenone, 3-ethylcyclopentanone, 5-methylohexan-3-one, benzophenone, 2-pentanone, 2-heptanone, 2-butanone, 3-hexanone, 4-heptanone, 3-methylcyclopentanone, cyclohexanone and 1-octen-3-one. The next groups were aldehydes and alcohols, ca. 10 and 12% of all the chemical compounds respectively. We observed the presence of a series of aliphatic aldehydes from acetaldehyde to decanal, including also unsaturated and methylated compounds (i.e., 2-methylbutanal, 2-methyl-2-butenal). This is similar to the observations of Smith and Ratclife [6,7]. Nonanal was identified in the majority of samples. We also found aldehydes containing a benzene ring, i.e., benzaldehyde, 2,4-dimethylbenzaldehyde, 2-hydroxybenzaldehyde, 4-methylbenzaldehyde, 4-(1-methylethyl)-benzaldehyde and alpha-methylbenzeneacetaldehyde. As for the presence of alcohols in the samples, the most frequently observed were 1-dodecanol, 1-octanol, 1-tetradecanol, benzyl alcohol, 1-hexanol, 1-nonanol, 1-octen-3-ol and 1-butanol. Many terpenes were identified among the detected substances. The substances most frequently present in the samples included limonene, pinene, menthadienes, mentol, mentone, valencene, geraniol, linalool, thujene, myrcene, sabinene hydrate, β-caryophyllene, linalool oxides, etc. Considering that terpenes are produced by plant organisms and not the human body, we assumed that the source of terpenes and their metabolites in urine is food. This assumption is supported also by the works of other [6,7]. Due to this fact, we excluded this group of substances from the set of potential disease markers.

Another exogenous group were plasticizers, most frequently diethyl phthalate and antioxidants (BHA, BHT), which we excluded from the analysis as they constitute an addition to polymers. Similarly, we removed silicones and oximes as these substances mostly come from polymers in urine containers, plastic tips, septa for HS vials etc. This is supported by blank analyses done according to the same procedure as urine sample analysis but containing only distilled water and NaCl. Phenol and cresols were found in urine samples, as well as dimethylphenols, guaiacol, 2-methoxy-4-vinylphenol and eugenol. Phenol and cresols are typical metabolites present in urine, identified by many authors. There is a correlation between the content of phenols in urine and the amount of consumed proteins [7].

A numerous group of substances were also N-, O-, S-heterocycles. The most frequently identified of them was indole and less common were 3-methylindole (skatole) as well as substituted pyridines, pyrroles, pyrazines, furans and benzothiazoles. The source of indole and skatole may be bacterial metabolism of aromatic amino acids (tyrosine, phenylalanine and tryptophan) occurring in the intestines. These substances may subsequently be absorbed into the blood and excreted with urine. We also observed series of gamma and delta lactones, i.e., nonalactone, decalactone and undecalactone as well as coumarin. However, the origin of such substances in urine has not been explained. Another group of chemicals are benzene and its alkyl derivatives (toluene, dimethyl benzenes, ethyl benzene, propylbenzene, styrene, etc.). On the one hand, these substances are known to be typical environmental pollutants; on the other, many studies consider them to be probable disease markers. We decided that we can overlook benzene, toluene and xylene isomers as exogenous substances. Nevertheless, isomers of trimetylbenzenes, ethylmethylbenzenes, naphtalene and its derivatives are included in our statistical analysis. Figure S1 shows structure ordered GC×GC chromatogram of urine (Supplementary Material).

Little is known, at present, whether the substances identified in the urine samples from ill and healthy people are created as a result of metabolism in the cells of a human organism, whether they originate from the diet or from metabolic changes occurring under the influence of an illness in the body. We observed elevated levels of benzeneacetaldehyde; 1-octanol; 1-decanol, 6-methylhept-5-en-2-one in urine of patients. Regarding the origin, phenol is very common metabolite existing in urine. Increased level of phenol can be explained by extensive metabolism of proteins or increasing of bacterial activity in the colon. 9-Octadecene-1-ol may be oxidation products of certain hydrocarbons conducted by cytochrome P450 or there are products of oxidative stress. 6,10-Dimethyl-5,9-undecadiene-2-one can be formed by the decarboxylation of keto acids generated during fatty acids metabolism. Their origin can be associated to oxidation of fatty acids. Moreover, inflammation processes are important factor in glomerular diseases. In this case a key role is played by cellular and humoral responses involving creating immuno complexes (circulating and in situ-formed) and complement pathways [21]. Oxidative stress and inflammation are initiated by the reactive oxygen species (ROS). Hence, ROS induce formation of several by products such as fatty acids, hydrocarbons, aldehydes and alcohols. The origin of the proposed markers can be connected mainly with oxidative stress. The knowledge regarding the biosynthesis of VOCs in the organism is very limited and covers only a small number of volatiles identified in bodily fluids and tissue. Parts of the substances present in urine are exogenous substances which enter the organism as food or flavors (terpenes), as well as environmental pollutants (aromatic hydrocarbons). Another group are exogenous substances that are transdermally absorbed into the organism, where they can be metabolized or not and then excreted with urine. At the moment there are studies to define new specific biomarkers of kidney damage, detected in both serum and urine. These include cystatin C, neutrophil gelatinase-associated lipocalin, kidney injury molecule-1 and interleukin 18 [21]. Nevertheless, our study proved the discrimination between two investigated groups (the group with glomerular diseases and the control group) was possible based on the VOCs released from urine samples. Moreover, 4 VOCs that presented statistically significant differences between the groups can be assumed as markers of glomerular diseases. These are significantly increased peaks area in the patients group and they can be assumed as direct chemo indicators for glomerular diseases, while the other four were significantly lower and they may be considered as secondary chemo indicators. However, our research should be treated as a preliminary study, as the number of persons participating in the study was too small to draw more unequivocal conclusions, but using the developed methodology and involving higher number of patients, more deep investigations will be realized, with respect of non-proliferative or proliferative types. This will make the object of another study.

## 4. Materials and Methods

### 4.1. Materials

SPME device as well as Carboxene/PDMS, PDMS/Divinylbenzene and PDMS coated fibers were purchased from Supelco (Bellefonte, PA, USA). The screw top headspace glass vials with silicon/PTFE septa and caps were supplied by Supelco. Sodium chloride was purchased from Sigma-Aldrich (Steinheim, Germany) and Sil Tite micro union from Trajan (Trajan, Rigwood Victoria, Australia) Ultrahigh purity helium BIP 5.5 was purchased from Air Liquide, Poland.

### 4.2. Apparatus

The analysis was carried out using a Leco Pegasus 4D GCGC TOF-MS instrument (Leco Corp., St. Joseph, NH, USA) equipped with a dual stage jet cryogenic modulator. The mass spectrometer was hyphenated with an Agilent 6890 gas chromatograph (Agilent Technologies, Waldbronn, Germany). The gas chromatograph was equipped with a PTV injector (Gerstel, Mulhheim, Germany) with 0.75 mm ID liner and MPS-2 autosampler, (Gerstel, Mulhheim, Germany) for automatic SPME extraction and fiber desorption. The first-dimension column was RTX-Wax capillary (30 m × 0.25 mm × 0.20 μm) integrated with deactivated guard column (5 m × 0.25 mm) and the second-dimension column was a 1.5 m Rtx–1 capillary (1.5 m × 0.18 mm × 0.20 µm), both supplied by Restek (Restek, Bellefonte, PA, USA). The first column was connected to the second analytical column with an SGE micro union. The injector temperature was kept at 230 °C. SPME desorptions were made in the splitless mode within 1 min. Helium was used as the carrier gas in constant flow mode at 1 mL/min. The mass spectrometer was operating as follows both ion source temperature and transfer line 225 °C; ionization energy 70 eV (electron impact ionization); acquisition range 35–350 amu; acquisition range 180 Hz. The first-dimension column temperature was programmed as follows: the initial temperature of 40 °C was held for 3 min and then the temperature was increased by 10 °C/min to 235 °C and maintained for 5 min at this value. The second-dimension column temperature was maintained 5 °C higher than the corresponding first dimension column. The modulator temperature was maintained 15 °C higher than the corresponding one of the second-dimension column and the modulation period was 3 s, hot 0.9 s and cold 0.6 s. The programming rate and hold times were the same for the two columns and modulator.

### 4.3. Data Processing

Data processing parameters were as follow tune check on, baseline offset 1, peak width 2 s. for 2-nd dimension, match spectra require to combine was 700, minimum S/N 11 subpeak to be retained, maximum number of unknown peaks to find 10,000, automatic peak finding S/N = 5, library search mode-forward, library identify-normal, maximum mass 350, library mass threshold 5, minimum similarity match 900, peak mass to use for area calculation-unique mass. For mass spectrometry QC method, the instrument was adjusted to optimization mass 219 m/z, with minimum intensity 20,000 and maximum intensity 100,000. Chromatof (Leco) software version 4.50.8.0 was used for data acquisition and processing.

### 4.4. Sample Pre-Processing and Extraction

Obtained urine samples were immediately frozen and stored at −20 °C. In this study, frozen samples were not stored longer than 5 days before analysis. Prior to analyses, the samples were defrosted and then immediately pre-processed and analyzed as follows. NaCl (3.6 g) was weighed in a 20 mL HS glass vial and 10.0 mL of urine sample was added. Then, the vial was capped with a PTFE septum and a screw cap. The sample vial was incubated for 30 min at 45 °C. The fiber was exposed to the headspace at 45 °C for 45 min. After sampling, the SPME fiber was withdrawn into the needle, removed from the vial and inserted into the GC injector port for 1 min in the splitless mode, wherein the metabolites were thermally desorbed and transferred directly into the column.

### 4.5. Human Subjects

The patients were recruited among the patients of Nephrology, Diabetology and Internal Medicine Department (Collegium Medicum, Nicolaus Copernicus University, Rydygier Hospital, Torun, Poland). We selected a population of 27 adult patients (14 female, 13 male) with confirmed glomerular diseases and enrolled healthy control volunteers (20 persons). The mean age was 48.0 years. One male patient was a smoker and 3 patients (15%) had kidney biopsy in the past. The patients and the controls were not restricted to any particular diet. Serum creatinine concentration ranged from 0.5 to 3.87 mg/dl (mean 1.26). Estimated glomerular filtration rate (eGFR) ranged from 15 to 126 mL/min/1.73 m^2^. Serum urea concentration ranged from 24 to 236 mg/dl (mean 58). C-reactive protein average was 9.1 mg/L (range 0.1–79). Early morning mid-stream urine samples were collected in 100 mL sterile plastic containers at hospital. Afterwards, samples were immediately frozen and stored at −20 °C. Prior to analyses, the samples were defrosted.

All subjects gave their informed consent for inclusion before they participated in the study. The study was conducted in accordance with the Declaration of Helsinki and the protocol was approved by the Ethics Committee of Collegium Medicum in Bydgoszcz (No. KB 621/2016-25.10.2016).

## 5. Conclusions

Our work focused on the development a methodology able to differentiate patients with glomerular diseases from healthy persons. Our study highlighted that volatile profiles coming from the two groups, diseased and controls can be easily discriminated by network analysis. Cluster analysis (based on Squared Euclidean distance) also segregated the two investigated groups. Dataset reduction highlighted that all persons with glomerular diseases had elevated level of several dozens of compounds with different origins; however, four compounds (methyl hexadecanoate; 9-hexadecen-1-ol; 6,10-dimethyl-5,9-undecadien-2-one, 2-pentanone were finally, proposed as markers of glomerular diseases. Identified compounds may be promising biomarker candidates for discrimination of patients with glomerular diseases and healthy volunteers. Moreover, deeper investigations of the biochemical pathways of the particular compounds as well selection of larger group of participants, with respect of non-proliferative or proliferative types, are essential. In the future perspective, investigations focused on person’s diet will be particularly interesting.

## Figures and Tables

**Table 1:** ANOVA grouping results of VOC in k-mean analysis (disease group vs. control). where: SS Group–squared sum, df-degree of freedom, SS Error-error sum of square, F-F test, p-probability value.

ID	Compound	SS_Group_	df	SS_Error_	df	*F*	*p*
1	Cyclohexanone	160.14	1	99.80	39	54.56	0.000
2	3-Ethylcyclopentanone	60.33	1	121.36	39	16.90	0.000
3	3-Hexanone	87.83	1	166.31	39	17.96	0.000
4	3-Heptanone	102.78	1	145.92	39	23.95	0.000
5	Methyl hexadecanoate	68.88	1	134.76	39	17.38	0.000
6	9-Hexadecen-1-ol	75.72	1	142.22	39	18.10	0.000
7	3-Methyl-2-pentanone	73.83	1	209.25	39	12.00	0.001
8	6,10-Dimethyl-5,9-undecadien-2-one	49.61	1	117.49	39	14.36	0.001
9	2-Pentanone	44.38	1	144.91	39	10.41	0.003
10	Acetophenone	25.60	1	117.55	39	7.40	0.010
11	2-Methoxy-4-vinylphenol	19.72	1	119.53	39	5.61	0.024
12	1-Decanol	28.56	1	184.65	39	5.26	0.028
13	N-Acetylpyrrole	28.44	1	204.56	39	4.73	0.037
14	6-Methylhept-5-en-2-one	22.96	1	198.97	39	3.92	0.056
15	Dimethyl sulfone	3.24	1	37.79	39	2.91	0.097
16	1-Tetradecanol	14.96	1	190.06	39	2.68	0.111
17	4-Heptanone	9.43	1	128.86	39	2.49	0.124
18	Benzaldehyde	1.66	1	23.90	39	2.36	0.134
19	2-Nonanone	14.35	1	213.06	39	2.29	0.139
20	5-Methyl-3-hexanone	13.07	1	210.41	39	2.11	0.155
21	Dimethyl trisulfide	10.23	1	190.57	39	1.83	0.186
22	2-Aminobenzaldehyde	9.40	1	222.11	39	1.44	0.239
23	3-Methylcyclopentanone	8.12	1	230.47	39	1.20	0.281
24	Hexanal	3.85	1	217.07	39	0.60	0.443
25	1-Octanol	3.59	1	245.37	39	0.50	0.485
26	Benzeneacetaldehyde	3.43	1	234.68	39	0.50	0.486
27	2,5-Dimethylpyrazine	2.85	1	198.49	39	0.49	0.490
28	Nonanal	1.50	1	155.73	39	0.33	0.571
29	9-Octadecen-1-ol	1.47	1	183.26	39	0.27	0.605
30	Indole	0.35	1	49.34	39	0.24	0.626
31	Theaspirane	1.37	1	218.11	39	0.21	0.647
32	Benzonitrile	0.52	1	185.47	39	0.09	0.760
33	2-Heptanone	0.27	1	157.55	39	0.06	0.811
34	4-Methylphenol	0.27	1	170.99	39	0.05	0.818
35	Phenol	0.24	1	166.54	39	0.05	0.828
36	Decanal	0.09	1	73.76	39	0.04	0.837
37	1-Methyl-4-(1-methylethenyl)-benzene	0.08	1	192.35	39	0.01	0.905
38	N-Phenylformamide	0.00	1	227.31	39	0.00	0.979
39	Ethyl acetate	0.00	1	328.20	39	0.00	0.986
40	Octanal	0.00	1	199.51	39	0.00	0.986

**Table 2:** Descriptive statistics of the VOCs defined in higher concentration in urine by patients suffering from glomerular diseases, where: min–minimum, max–maximum.

		Diseased Group			Healthy Group		
ID	Compound	Mean	Min	Max	Mean	Min	Max
1	Cyclohexanone	3.28 × 10^5^	1.33 × 10^4^	9.43 × 10^5^	3.54 × 10^4^	2.23 × 10^4^	4.64 × 10^4^
2	3-Ethylcyclopentanone	1.10 × 10^5^	5.14 × 10^3^	4.53 × 10^5^	3.41 × 10^4^	9.57 × 10^3^	6.58 × 10^4^
3	3-Hexanone	9.41 × 10^5^	1.65 × 10^4^	1.35 × 10^6^	3.44 × 10^4^	2.92 × 10^4^	1.06 × 10^6^
4	3-Heptanone	2.84 × 10^6^	6.06 × 10^4^	2.57 × 10^7^	1.75 × 10^5^	7.46 × 10^4^	3.90 × 10^6^
5	Methyl hexadecanoate	7.63 × 10^4^	7.64 × 10^3^	3.64 × 10^5^	2.19 × 10^4^	6.99 × 10^3^	8.38 × 10^4^
6	9-Hexadecen-1-ol	7.39 × 10^4^	5.28 × 10^3^	4.34 × 10^5^	9.39 × 10^3^	2.78 × 10^3^	1.93 × 10^5^
7	3-Methyl-2-pentanone	2.69 × 10^6^	2.90 × 10^4^	2.81 × 10^7^	1.81 × 10^5^	8.95 × 10^3^	1.19 × 10^7^
8	6,10-Dimethyl-5,9-undecadien-2-one	1.41 × 10^5^	2.47 × 10^4^	3.91 × 10^5^	7.41 × 10^4^	1.93 × 10^4^	1.73 × 10^5^
9	2-Pentanone	1.94 × 10^7^	6.39 × 10^4^	7.62 × 10^7^	3.18 × 10^6^	1.46 × 10^6^	1.05 × 10^8^
10	Acetophenone	1.08 × 10^5^	4.37 × 10^4^	1.40 × 10^5^	1.23 × 10^4^	8.19 × 10^4^	1.79 × 10^5^
11	2-Methoxy-4-vinylphenol	1.95 × 10^5^	1.76 × 10^4^	9.97 × 10^5^	1.07 × 10^5^	1.19 × 10^4^	4.58 × 10^5^
12	1-Decanol	1.62 × 10^5^	2.46 × 10^4^	5.20 × 10^5^	2.82 × 10^5^	1.99 × 10^4^	7.00 × 10^5^
13	N-Acetylpyrrole	3.12 × 10^5^	1.11 × 10^4^	1.06 × 10^6^	6.87 × 10^4^	3.17 × 10^4^	7.43 × 10^5^
14	6-Methylhept-5-en-2-one	1.84 × 10^5^	9.19 × 10^3^	9.31 × 10^5^	2.00 × 10^5^	2.30 × 10^4^	7.26 × 10^5^
15	Dimethyl sulfone	3.24 × 10^4^	1.10 × 10^4^	1.97 × 10^5^	2.97 × 10^4^	4.16 × 10^3^	9.29 × 10^4^
16	1-Tetradecanol	2.93 × 10^5^	1.33 × 10^4^	1.23 × 10^6^	2.11 × 10^5^	3.10 × 10^4^	4.69 × 10^5^
17	4-Heptanone	6.50 × 10^6^	2.24 × 10^4^	3.82 × 10^7^	5.73 × 10^6^	1.77 × 10^4^	2.25 × 10^7^
18	Benzaldehyde	6.23 × 10^4^	6.18 × 10^3^	1.89 × 10^5^	1.01 × 10^5^	1.26 × 10^4^	3.58 × 10^5^
19	2-Nonanone	1.56 × 10^5^	1.31 × 10^4^	7.72 × 10^5^	1.54 × 10^5^	1.07 × 10^4^	5.24 × 10^5^
20	5-Methyl-3-hexanone	1.89 × 10^6^	5.78 × 10^4^	1.48 × 10^7^	3.22 × 10^6^	1.92 × 10^4^	1.90 × 10^7^
21	Dimethyl trisulfide	6.17 × 10^4^	5.14 × 10^3^	1.88 × 10^5^	4.51 × 10^4^	5.76 × 10^3^	7.46 × 10^4^
22	2-Aminobenzaldehyde	1.93 × 10^5^	6.12 × 10^3^	6.80 × 10^5^	2.65 × 10^5^	8.50 × 10^4^	5.20 × 10^5^
23	3-Methylcyclopentanone	2.76 × 10^5^	4.29 × 10^4^	8.61 × 10^5^	2.05 × 10^5^	3.19 × 10^4^	7.21 × 10^5^
24	Hexanal	1.80 × 10^6^	1.16 × 10^5^	6.48 × 10^6^	9.01 × 10^5^	8.86 × 10^4^	1.86 × 10^6^
25	1-Octanol	5.13 × 10^5^	1.01 × 10^4^	3.61 × 10^6^	6.37 × 10^5^	7.98 × 10^4^	3.42 × 10^6^
26	Benzeneacetaldehyde	1.43 × 10^5^	2.67 × 10^4^	1.25 × 10^6^	1.47 × 10^5^	5.34 × 10^4^	5.60 × 10^5^
27	2,5-Dimethylpyrazine	1.19 × 10^5^	1.06 × 10^4^	5.67 × 10^5^	7.50 × 10^4^	1.82 × 10^4^	2.36 × 10^5^
28	Nonanal	2.26 × 10^5^	2.04 × 10^4^	1.09 × 10^6^	1.28 × 10^5^	7.82 × 10^4^	1.77 × 10^5^
29	9-Octadecen-1-ol	4.98 × 10^5^	3.25 × 10^4^	1.79 × 10^6^	4.60 × 10^5^	3.20 × 10^4^	9.43 × 10^5^
30	Indole	1.20 × 10^5^	2.19 × 10^4^	3.47 × 10^5^	9.08 × 10^4^	1.45 × 10^4^	2.00 × 10^5^
31	Theaspirane	1.09 × 10^5^	7.87 × 10^3^	2.62 × 10^5^	1.29 × 10^5^	4.64× 10^4^	3.72 × 10^5^
32	Benzonitrile	4.53 × 10^4^	6.57 × 10^3^	6.30 × 10^4^	1.67 × 10^4^	8.76 × 10^3^	3.10 × 10^4^
33	2-Heptanone	1.92 × 10^5^	1.13 × 10^4^	1.41 × 10^6^	1.75 × 10^5^	7.46 × 10^4^	5.69 × 10^5^
34	4-Methylphenol	1.22 × 10^5^	1.71 × 10^4^	5.13 × 10^5^	1.71 × 10^5^	2.71 × 10^4^	1.18 × 10^6^
35	Phenol	3.52 × 10^4^	9.95 × 10^3^	4.67 × 10^5^	2.90 × 10^4^	2.08 × 10^4^	1.11 × 10^5^
36	Decanal	1.39 × 10^5^	1.62 × 10^4^	7.38 × 10^5^	1.56 × 10^4^	2.06 × 10^4^	2.06 × 10^4^
37	1-Methyl-4-(1-methylethenyl)-benzene	1.13 × 10^5^	1.12 × 10^4^	1.41 × 10^5^	1.41 × 10^5^	1.90 × 10^4^	6.09 × 10^5^
38	N-Phenylformamide	1.17 × 10^5^	1.59 × 10^4^	3.32 × 10^5^	1.54 × 10^5^	3.02 × 10^4^	3.35 × 10^5^
39	Ethyl acetate	1.56 × 10^7^	2.02 × 10^5^	7.11 × 10^7^	1.41 × 10^7^	1.21 × 10^1^	6.41 × 10^57^
40	Octanal	2.17 × 10^5^	5.62 × 10^4^	6.97 × 10^5^	1.62 × 10^5^	1.49 × 10^5^	4.34 × 10^5^

## Data Availability

Data is contained within the article.

## Supplementary Materials

The following are available online at https://www.mdpi.com/1420-3049/26/7/1817/s1, Figure S1: structure ordered GCxGC chromatogram of urine sample from patient. Peak groups: 1—Ketones (2-pentanone, 3-methyl-2-pentanone, 3-hexanone, 3-heptanone, 5-methyl-3-hexanone, acetophenone), 2—Aldehydes (hexanal, heptanal, octanal, nonanal, decanal, benzaldehyde, benzeneacetaldehyde), 3—Alcohols (1-hexanol, 1-octanol, 1-nonanol, menthadienol, 1-decanol, verbenol, 1-dodecanol, 1-tetradecanol, 9-hexadecen-1-ol, 9-octadecen-1-ol), 4—Phenols (phenol, 4-methylphenol).

## Sample Availability

Samples of the compounds are not available from the authors.
