# Supplementary material for: Searching for Potential Markers of Glomerulopathy in Urine by HS-SPME-GC×GC TOFMS
Source: Molecules. 2021 Mar 24;26(7):1817. doi: 10.3390/molecules26071817 (PMC8036251; doi:10.3390/molecules26071817)
Supplement: Supplementary file 1 [file molecules-26-01817-s001.pdf]

Supplementary Material for

"Searching for Potential Markers of Glomerulopathy in Urine by HS-SPME-GC×GC TOFMS"

Tomasz Ligor [1,2,*], Joanna Zawadzka [3], Grzegorz Strączyński [4], Rosa M. González Paredes [5], Anna Wenda-Piesik [6], Ileana Andreea Ratiu [7,2] and Marek Muszytowski [3]

1 Department of Environmental Chemistry and Bioanalytics, Faculty of Chemistry, Nicolaus Copernicus University, 87-100 Toruń, Poland;
2 Interdisciplinary Centre of Modern Technologies, Nicolaus Copernicus University, 87-100 Toruń, Poland; andreea_ratiu84@yahoo.com
3 Department of Nephrology, Diabetology and Internal Medicine, Nicolaus Copernicus University, Rydygier Hospital, 87-100 Toruń, Poland; as.zawadzka@gmail.com (J.Z.); marek.muszytowski@gmail.com (M.M.)
4 USL Ltd., 43-110 Tychy, Poland; grzegorz.straczynski@usl.com.pl
5 Department of Analytical Chemistry, Nutrition and Food Sciences, University of Salamanca,
37008 Salamanca, Spain; rosamgonzal@usal.es
6 Department of Plant Growth Principles and Experimental Methods, UTP University of Science and Technology, 85-796 Bydgoszcz, Poland; apiesik@utp.edu.pl
7 "Raluca Ripan" Institute for Research in Chemistry, Babes-Bolyai University, 30 Fantanele, RO-400239 Cluj Napoca, Romania;
* Correspondence: tligor@umk.pl

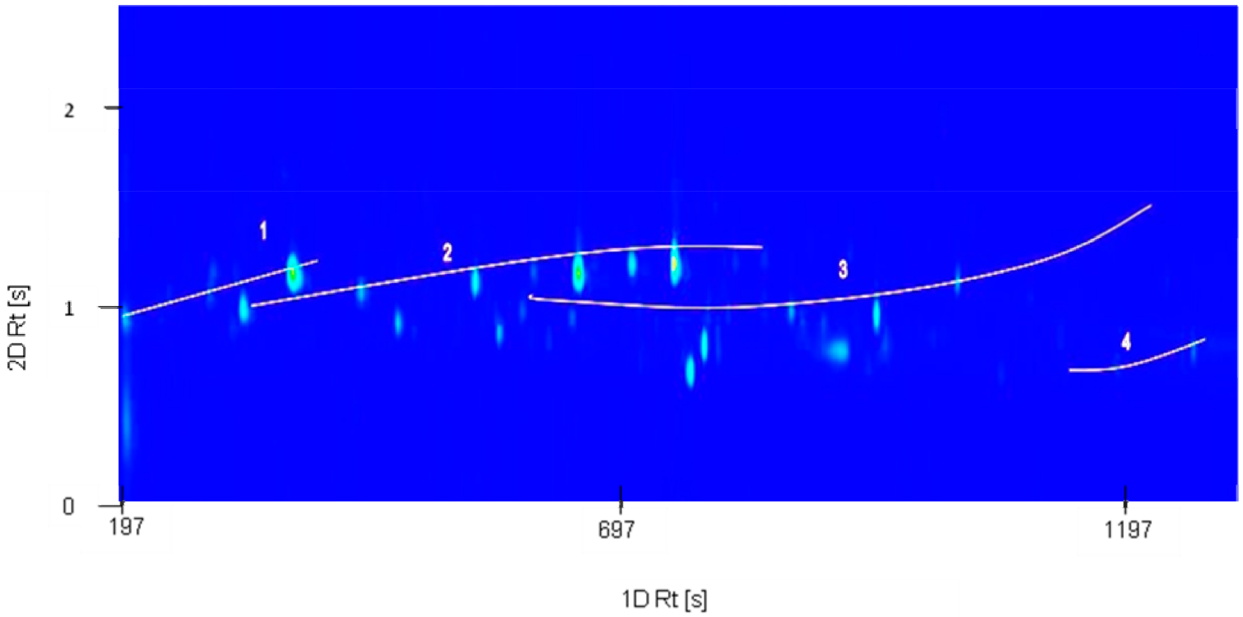

Figure S1. Structure ordered GCxGC chromatogram of urine sample from patient. Peak groups: 1 - Ketones (2-pentanone, 3-methyl-2-pentanone, 3-hexanone, 3-heptanone, 5-methyl-3-hexanone, acetophenone), 2 - Aldehydes (hexanal, heptanal, octanal, nonanal, decanal, benzaldehyde, benzeneacetaldehyde), 3 - Alcohols (1-hexanol, 1-octanol, 1-nonanol, menthadienol, 1-decanol, verbenol, 1-dodecanol, 1-tetradecanol, 9-hexadecen-1-ol, 9-octadecen-1-ol), 4 – Phenols (phenol, 4-methylphenol).
